# Supplementary material for: Prognostic Value of Albumin to Globulin Ratio in Non-Metastatic and Metastatic Prostate Cancer Patients: A Meta-Analysis and Systematic Review
Source: Int J Mol Sci. 2022 Sep 29;23(19):11501. doi: 10.3390/ijms231911501 (PMC9570150; doi:10.3390/ijms231911501)
Supplement: Supplementary file 1 [file ijms-23-11501-s001.zip › Supplementary Table S1.pdf]

Supplementary Table S1. Risk of Bias for all studies included in the meta-analysis.

RISK OF BIAS				
	**Patient selection**	**index tests**	**Reference standard**	**Flow and timing**
**Chung JM et al (20)**	LOW	UNCLEAR	LOW	LOW
**Quhal F et al (21)**	LOW	LOW	UNCLEAR	LOW
**Aydh A et al (19)**	LOW	LOW	UNLCEAR	LOW
**Wang et al (18)**	LOW	LOW	LOW	UNCLEAR
