# Supplementary material for: Prognostic Value of Albumin to Globulin Ratio in Non-Metastatic and Metastatic Prostate Cancer Patients: A Meta-Analysis and Systematic Review
Source: Int J Mol Sci. 2022 Sep 29;23(19):11501. doi: 10.3390/ijms231911501 (PMC9570150; doi:10.3390/ijms231911501)
Supplement: Supplementary file 1 [file ijms-23-11501-s001.zip › Title page AGR.pdf]

PROGNOSTIC VALUE OF ALBUMIN TO GLOBULIN RATIO IN NON-METASTATIC AND METASTATIC PROSTATE CANCER PATIENTS: A META-ANALYSIS AND SYSTEMATIC REVIEW

Stefano Salciccia [a], Marco Frisenda [a] , Giulio Bevilacqua [a] , Pietro Viscuso [a], Paolo Casale [b] ,Ettore De Berardinis [a], Giovanni Battista Di Pierro [a], Susanna Cattarino [a], Gloria Giorgino [a], Davide Rosati [a], Francesco Del Giudice [a] , Alessandro Sciarra [a] , Gianna Mariotti [a], Alessandro Gentilucci [a]

Affiliations:

[a] Department of Maternal-Infant and Urologic Sciences, 'Sapienza' University of Rome, Policlinico Umberto I Hospital, Rome, Italy.

[b] Department of Urology, Humanitas, Milan, Italy

All authors listed gave a substantive contribution to this study and to this original article

Running title: Meta-analysis on albumin to globulin ratio

Words count: 2293 (243 words Abstract); Tables: 1; Figures: 2; Supplementary Figures: 2; Supplementary Table :1

Key words: prostatic neoplasm, albumin to globulin ratio, meta-analysis, radical prostatectomy, hormone therapy

Corresponding Author:

Prof Alessandro Sciarra, MD

Department of Maternal-Infant and Urologic Sciences, 'Sapienza' University of Rome. Policlinico Umberto I Hospital, Viale del Policlinico 151, Rome, 00161, Italy

Phone: +39-0649977000; Email: alessandro.sciarra@uniroma1.it
