# Supplementary figures and images for: Prognostic Value of Albumin to Globulin Ratio in Non-Metastatic and Metastatic Prostate Cancer Patients: A Meta-Analysis and Systematic Review
Source: Int J Mol Sci. 2022 Sep 29;23(19):11501. doi: 10.3390/ijms231911501 (PMC9570150; doi:10.3390/ijms231911501)

Galbraith plot

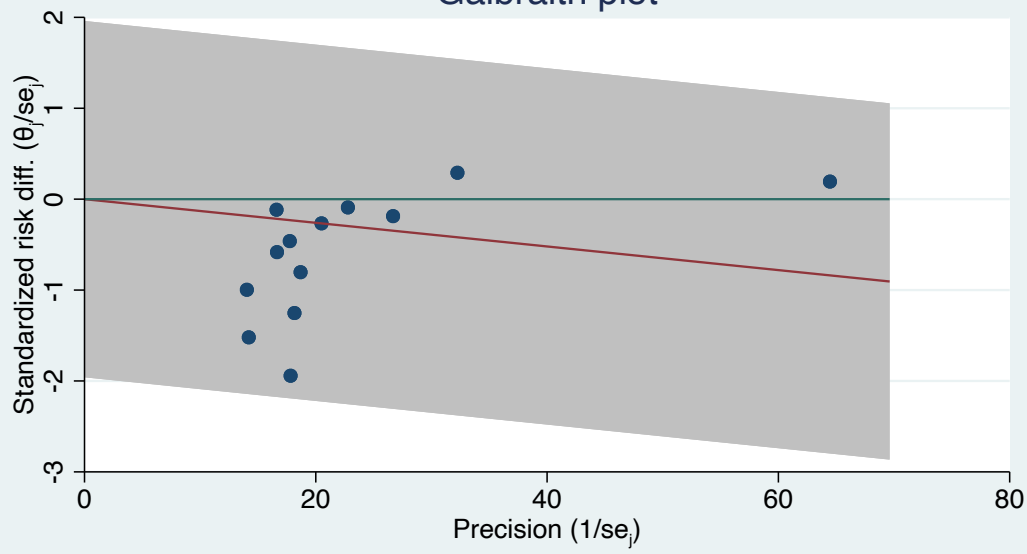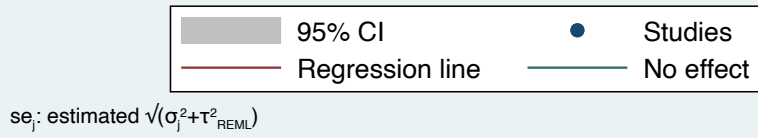

Supplement: Supplementary file 1 [file ijms-23-11501-s001.zip › Supplementary Figure S1 AGR.pdf]

Funnel plot

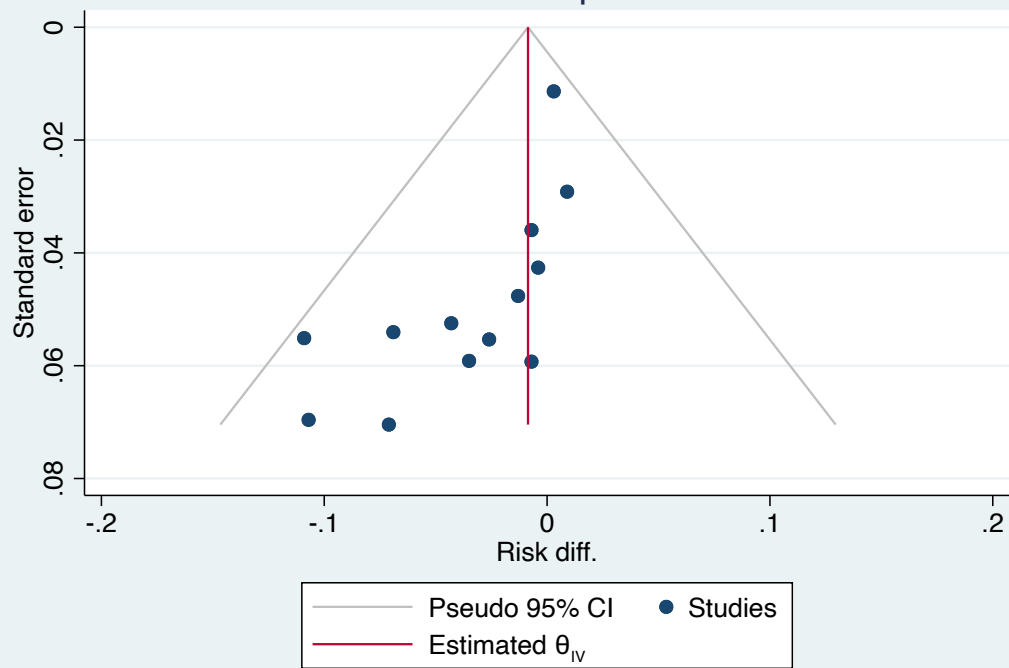

Supplement: Supplementary file 1 [file ijms-23-11501-s001.zip › Supplementary Figure S2 AGR.pdf]
